# Advances in the treatment of hepatogenous diabetes: A review

**DOI:** 10.1097/MD.0000000000036068

**Published:** 2023-11-17

**Authors:** Yanru Deng, Keyu Li, Ang Li, WeiMing Hu, Wen Hu

**Affiliations:** a Department of Clinical Nutrition, West China Hospital; Sichuan University, Chengdu, Sichuan Province, China; b Division of Pancreatic Surgery, Department of General Surgery, West China Hospital; Sichuan University, Chengdu, Sichuan Province, China.

**Keywords:** cirrhosis, hepatogenous diabetes, insulin resistance, liver disease, oral antidiabetic drug, type 2 diabetes

## Abstract

Hepatogenous diabetes (HD) is a glycogen metabolism disorder that arises as a consequence of chronic liver disease. The condition is frequently detected in patients diagnosed with cirrhosis, which is a result of advanced liver disease. The prognosis for patients with HD is generally poor, and they are at a heightened risk for serious complications such as gastrointestinal bleeding, primary peritonitis, and hepatic encephalopathy. Hepatogenous diabetes progression is often associated with cirrhosis progression, which leads to the development of liver cancer and increased patient mortality. Despite the prevalence and severity of HD, no systematic treatment strategy for clinical management of the condition has been proposed by any research or institutions to date. This paper conducts an extensive review of recent advancements in HD treatment in the quest for an effective treatment approach that may improve the overall prognosis of HD.

## 1. Introduction

The liver performs a crucial function in regulating glucose homeostasis, which involves the control of various glucose metabolic pathways, such as glycolysis, glycogenolysis, gluconeogenesis, and glycogenesis.^[1]^ During fasting, the liver generates glucose through a series of biochemical processes, with excess glucose being stored as glycogen upon saturation of glucose consumption.^[2]^ However, chronic liver diseases such as hepatitis and cirrhosis disrupt the liver glycogen metabolism system, leading to the onset of hepatogenous diabetes (HD).^[3]^

Hepatogenous diabetes shares similarities with diabetes mellitus, particularly type 2 diabetes mellitus (T2DM). Nevertheless, HD patients typically lack a history of diabetes or family history but have a history of chronic liver disease prior to diabetes onset. In some cases, HD patients may exhibit symptoms of both diabetes and liver disease.^[4]^ What's worse, the coexistence of hyperglycemic state and primary liver disorders such as cirrhosis in HD patients increases the risk of severe complications, thereby resulting in a vicious cycle.^[5–7]^ Consequently, differential diagnosis of HD and T2DM is essential in clinical management.^[3]^ The degree of liver fibrosis positively correlates with the severity of HD but not with the common risk factors for T2DM. Clinical manifestations of HD are primarily associated with liver disease, and the typical symptoms of primary diabetes such as polydipsia, polyphagia, polyuria, and weight loss are less frequently observed.^[8]^ Although both HD and T2DM exhibit insulin resistance, the degree of insulin resistance in HD patients is significantly higher compared to those with T2DM.^[9]^ Nevertheless, adequate treatment of HD can gradually overcome the original metabolic abnormality.^[10]^

### 1.1. Pathogenesis of HD

The pathogenesis of HD is not fully comprehended; however, it is known that insulin resistance and pancreatic β-cell dysfunction are the main factors leading to HD in cirrhosis patients.^[11]^ The following mechanisms may be responsible for the onset and progression of HD:

#### 1.1.1. Hyperinsulinemia and insulin resistance.

Firstly, damage to liver cells can reduce the physiological activity of the liver, thereby decreasing glycogen synthesis and impairing the liver capacity to inactivate insulin. This phenomenon has been linked to the occurrence of hyperinsulinemia.^[12]^ liver cirrhosis has been shown to lead to a decrease in insulin receptors, which can result in reduced insulin uptake by the liver. Furthermore, the decreased activity of liver enzymes can impair the liver capacity to inactivate glucagon, which can reduce insulin sensitivity. Moreover, portal vein collateral circulation can cause insulin to bypass the liver and enter into the systemic circulation directly. This, in turn, can affect the insulin inactivation pathway, leading to hyperinsulinemia and insulin resistance.^[13]^ The mechanisms of these processes are complex and interrelated, requiring further investigation to fully understand the underlying causes and potential treatments for these conditions.

#### 1.1.2. Dysfunction of pancreatic β-cells.

In patients with cirrhosis, despite increased production of insulin from pancreatic β-cell stimulation leading to hyperinsulinemia, insulin secretion is significantly reduced. This reduction may be due to changes in pancreatic congestion caused by liver fibrosis, vascular resistance, and portal hypertension. Additionally, chronic hyperglycemia causes toxic damage to the islets resulting in dysfunctional β-cells. The systemic hypoxia associated with advanced cirrhosis further exacerbates the β-cell dysfunction.^[14,15]^

#### 1.1.3. Hepatitis virus theory.

The hepatitis virus can directly invade the pancreas leading to dysfunction of the pancreatic β-cells, reduced insulin secretion, and elevated blood glucose levels.^[16]^ Additionally, the genes of hepatitis virus can combine with islet genes and result in the synthesis of denatured insulin with insulin phenotypes competing with normal insulin for inhibition. Furthermore, the hepatitis C virus can cause insulin resistance by destroying the insulin receptor substrate-1.^[17]^

#### 1.1.4. Nutritional factors.

Malnutrition resulting from chronic liver disease can lead to the degradation of pancreatic β-cells, impair insulin synthesis and secretion, and reduce peripheral insulin sensitivity. Prolonged intravenous infusion of glucose during the treatment process can overstimulate islet β-cells, decreasing their function and increasing the risk of failure.^[18,19]^ Intestinal dysbiosis, hyperammonemia, sarcopenia, and other factors may also contribute to the pathogenesis of HD.^[11]^

### 1.2. Treatment of HD

At present, there are no established clinical management guidelines for HD. In clinical practice, the treatment of HD shares similarities with that of type 2 DM. Natural remedies, such as low-calorie diets and physical exercise, should be planned according to patients’ liver function and blood glucose levels. Moreover, HD is a secondary condition that can be controlled by selecting appropriate oral antidiabetic drugs, including metformin, sulfonylureas, thiazolidinediones, alpha-glucosidase inhibitors, glucagon-like peptide-1 receptor agonists, and sodium-glucose co-transporter 2 inhibitors. Insulin injection is currently considered the safest and most effective method for the clinical treatment of HD. In addition, surgical treatments such as liver transplantation is an option for patients who do not respond to the aforementioned therapies.

#### 1.2.1. Natural remedies.

Natural remedies to manage T2DM include low-calorie diets and physical exercise. The primary objective of the former is to relieve the workload on pancreatic β-cells, while the latter aims to enhance peripheral insulin sensitivity. However, in patients with hepatic dysfunction, intermittent or low-calorie diets may exacerbate symptoms of malnutrition, weakness, emaciation, and even syncope or shock. Therefore, clinicians need to calculate the total daily calorie intake based on blood glucose levels and recommend a suitable combination of foods to decrease fat and high-energy intake, increase fruit and vegetable consumption, and maintain blood glucose levels. Moreover, moderate exercise can stimulate the synthesis of insulin receptors and improve insulin sensitivity. However, exercise in patients with hepatic dysfunction must be supervised, with proper guidance on exercise timing, methods, and rest periods to allow the liver to rest and promote its recover.^[20]^ Patients with inadequate glycemic control should be mindful of the intensity of exercise and carry energy bars to avoid sudden hypoglycemia.^[21]^ In summary, individualized comprehensive management for hepatic dysfunction-related diabetes should encompass personalized interventions in diet, exercise, hypoglycemic medications, and insulin use.

#### 1.2.2. Oral antidiabetic drugs (OAD).

The treatment plan for primary diabetes varies based on its different types. Generally, patients with type 1 diabetes are advised to use insulin throughout their lives, while patients with T2DM should improve insulin resistance by enhancing their physical health. Hepatogenous diabetes is categorized as secondary diabetes, and effective management of blood glucose levels during the course of the disease is crucial in preventing or delaying the onset of severe complications associated with diabetes.^[22]^ When treating HD patients with oral antidiabetic drugs, the target glycemic control should be geared towards postprandial blood glucose levels instead of the usual measures such as fasting blood glucose or glycated hemoglobin (HbA1c). Moreover, serum fructosamine can serve as an effective monitoring tool for the long-term glycemic control of HD patients, reflecting their blood glucose status for the past 2–4 weeks, and may be more reliable than HbA1c.^[23]^ Selecting the appropriate OADs for HD patients is challenging due to the underlying pathological and physiological changes caused by chronic liver diseases such as cirrhosis. These changes, along with altered pharmacokinetics of OADs, may heighten the risk of adverse events. Hence, it is vital to exercise prudence when selecting and combining drugs to avoid using medications with abnormal liver metabolism, low plasma protein binding, short half-life, or liver toxicity.^[24]^ Combination therapy may help enhance glycemic control in HD patients; however, it is crucial to carefully select and combine medications to prevent unfavorable outcomes.^[25]^ In instances where glycemic control remains inadequate, or liver function declines during the use of OADs, insulin therapy is considered a viable alternative for treatment.

##### 1.2.2.1. Metformin.

Metformin is a commonly used medication in clinical practice, owing to its numerous advantages such as high efficacy, a favorable safety profile, potential cardiovascular and metabolic benefits, and the ability to be used in combination with other antidiabetic agents.^[26]^ The glucose-lowering effect of metformin is primarily attributed to its ability to reduce liver gluconeogenesis and glycogen breakdown, enhance the uptake and utilization of glucose in peripheral tissues, promote insulin receptor binding, thus leading to improved glycemic control.^[27,28]^ In addition, Metformin has been found to reduce the risk of liver cancer and slow hepatic encephalopathy progression in individuals with diabetes and liver cirrhosis. Chen et al reported a 7% yearly reduction in the risk of primary liver cancer progression in individuals with liver cirrhosis who used metformin.^[29]^ A retrospective study of 82 patients with diabetes secondary to liver cirrhosis revealed a significant reduction in the probability of hepatic encephalopathy occurrence with metformin use, which may be attributed to its ability to inhibit glutamine synthetase activity.^[30]^ However, there have been concerns over the potential risk of lactic acidosis associated with metformin use, particularly in individuals with liver cirrhosis, and caution has been advised.^[31]^ Nevertheless, a systematic review and meta-analysis of 194 randomized controlled trials found no significant association between metformin and lactic acidosis. Furthermore, individuals who received metformin treatment had a longer median survival time compared to those who did not.^[32,33]^ Additionally, metformin is relatively inexpensive and poses a low risk of hypoglycemia; however, mild gastrointestinal dysfunction may occur, particularly in sustained-release formulations. Therefore, it is recommended that metformin may be used for blood glucose control in patients with diabetes and liver cirrhosis except in cases of severe metabolic disorders or respiratory dysfunction.^[34]^

##### 1.2.2.2. Sulfonylureas (SUs).

Sulfonylureas constitute one of the earliest and most commonly prescribed classes of antidiabetic drugs. This drug class includes well-known agents such as glipizide, glimepiride, gliclazide, and glyburide, among others. Sulfonylureas exert their clinical effects by targeting the sulfonylurea receptor located on the pancreatic beta cells, prompting closure of the adenosine triphosphate-regulated potassium channels. This, in turn, facilitates calcium influx and leads to insulin secretion. By overall effect, SUs lower blood glucose levels in patients. Sulfonylureas undergo extensive metabolism in the liver, exhibit wide serum protein binding, and over 50% are typically excreted via the kidneys. Notably, the pharmacokinetics of glipizide remain similar between individuals with chronic liver disease and healthy subjects. However, in clinical practice, SUs are typically avoided for patients with liver cirrhosis. This caution arises because these patients exhibit decreased drug inactivation, and hypoalbuminemia can potentiate the concentration of unbound SUs, thus heightening the risk of hypoglycemia.^[35]^ Additionally, while most SUs get adequately metabolized and excreted in healthy individuals, elderly or liver-/kidney-impaired patients may experience drug accumulation, further burdening liver/kidney function and worsening their underlying disease.^[36]^

##### 1.2.2.3. Thiazolidinediones (TZDs).

Thiazolidinediones represent agonists of peroxisome proliferator-activated receptors, contributing to such benefits as mild reductions in hepatic glucose output, attenuation of inflammatory factor activation, and raised tissue insulin sensitivity.^[37]^ Thiazolidinediones undergo extensive metabolism in the liver, with subsequent elimination via bile and feces. Overall, overweight or obese patients with a body mass index over 30 can benefit from TZD therapy.^[38]^ Of all the TZDs available, pioglitazone is the most commonly used one, exhibiting good liver safety. However, little investigation exists concerning its pharmacokinetics regarding liver cirrhosis.^[39]^ Yet, a meta-analysis of 8 randomized controlled trials involving patients with advanced liver fibrosis suggests that TZDs can inhibit collagen production by hepatic stellate cells, potentially slowing the progression of liver fibrosis.^[40]^ In light of such evidence, the clinical application of pioglitazone is rationally considered safer. Nonetheless, clinicians should maintain a lower dosage for patients with chronic liver disease (with a ceiling of 30 mg/day) and closely monitor liver function during treatment.^[41]^

##### 1.2.2.4. α-glucosidase inhibitors (AGIs).

α-glucosidase inhibitors such as acarbose are commonly used oral hypoglycemic agents in clinical settings. α-glucosidase inhibitors act in the gastrointestinal tract by inhibiting the activity of α-glucosidase, thereby reducing the reabsorption of glucose in the intestine.^[42]^ α-glucosidase inhibitors exhibit low systemic bioavailability, and their metabolism in the liver is almost non-existent, making them a relatively safe, effective, and well-tolerated treatment option for patients with chronic liver disease.^[43]^ α-glucosidase inhibitors delay the digestion and absorption of carbohydrates, reducing postprandial hyperglycemia without causing malnutrition. In a randomized double-blind study that included 100 patients with compensated cirrhosis and diabetes, treatment with acarbose significantly improved postprandial and fasting blood glucose levels.^[44]^ Furthermore, acarbose can promote the proliferation of saccharolytic bacteria, stimulate intestinal peristalsis, and reduce blood ammonia levels.^[43]^ Nevertheless, studies have indicated the potential for mild and transient hepatitis caused by acarbose, and liver function tests should be monitored regularly in patients with hepatic diseases.^[45]^ Compared to older AGIs, a new generation of AGIs like miglitol can be absorbed into the bloodstream through the upper small intestine after entering the digestive tract. Due to its structure being more similar to glucose, miglitol exhibits enhanced inhibition of glucose absorption. Moreover, miglitol is not metabolized by the liver, meaning that liver function impairment does not affect its pharmacokinetics.^[46]^

##### 1.2.2.5. Glucagon-like peptide-1 (GLP-1) receptor agonists.

Glucagon-like peptide-1 is a polypeptide hormone produced by intestinal L cells that accelerates glucose absorption by cells, promotes insulin secretion by pancreatic cells, and inhibits glucagon secretion by pancreatic alpha cells, thereby lowering blood glucose levels. In recent years, GLP-1 receptor agonists have gained popularity in the treatment of T2DM. Compared to traditional metformin drugs, GLP-1 receptor agonists are effective in lowering blood glucose levels and have other beneficial effects, such as protecting cardiovascular and cerebrovascular health, reducing body weight, and improving blood lipid levels.^[47,48]^

Liraglutide, a GLP-1 analog, not only exhibits some of GLP-1 functions but also regulates plasma superoxide dismutase and glycated serum protein levels, thus exhibiting certain antioxidant properties.^[49]^ Clinical studies have demonstrated that treating 52 patients with nonalcoholic fatty liver disease with liraglutide can inhibit the breakdown of fat tissue and the production of liver glycogen, thereby slowing the progression of liver fibrosis. Dulaglutide, a similar drug, also exhibits similar effects.^[50]^ Slowing the progression of liver fibrosis can extend the time before patients with liver cirrhosis enter the end-stage, thus prolonging their survival time. A pharmacokinetic study showed that taking liraglutide did not exacerbate the severity of liver damage in patients with varying degrees of liver injury, and the drug was well-tolerated.^[51]^ However, the use of GLP-1 receptor agonists in clinical settings is currently limited to Child-Pugh A patients, and caution is warranted when considering these drugs for patients with Child-Pugh B and C ratings.^[52]^

##### 1.2.2.6. Sodium-glucose co-transporter 2 (SGLT2) inhibitors.

Sodium-glucose co-transporter 2 inhibitors, including dapagliflozin, canagliflozin, and empagliflozin, have been found to reduce blood glucose levels by inhibiting the function of SGLT2 located in the proximal renal tubules. This inhibition ultimately leads to a decrease in the reabsorption of glucose by the renal tubules and encourages the excretion of glucose through urine. Numerous epidemiological studies have revealed a positive association between HbA1c levels and an increased risk of developing various cancers, particularly colon and liver cancers.^[53]^ In a study conducted by Crunkhorn, it was observed that SGLT2 transporters are expressed in certain tumors, which suggests that SGLT2 transporters may contribute to the proliferation of cancer cells by regulating their uptake and transportation of glucose.^[54]^ Patients with end-stage liver disease are often accompanied by liver fibrosis, which has a high probability of turning into liver cancer.^[55]^ Shiba and her colleagues discovered that compared with the control group, the incidence of nonalcoholic fatty liver disease-related liver cancer cases significantly reduced in the canagliflozin group, and there was also a noticeable decline in tumor volume. This result indicates that canagliflozin might delay the progression of nonalcoholic fatty liver disease into liver cancer.^[56]^ Sodium-glucose co-transporter 2 inhibitors possess the ability to protect kidneys, reduce water and sodium retention, and promote water excretion which makes them an excellent choice for patients with liver cirrhosis and simultaneous diabetes with ascites.^[57]^

#### 1.2.3. Insulin.

Insulin injection therapy is currently considered the safest and most effective way for the clinical management of HD. A study involving 348 patients with hepatitis C-related cirrhosis demonstrated that insulin therapy for secondary diabetes could achieve valuable therapeutic effects.^[55]^ Insulin primarily undergoes metabolism within the liver, and impaired liver function does not affect the pharmacokinetics of exogenous insulin or insulin analogs. The insulin requirement for cirrhosis patients may vary and will be difficult to predict due to reduced gluconeogenesis and insulin clearance rate in the liver; however, insulin resistance may increase.^[58]^ In cirrhosis patients using insulin therapy, close monitoring of blood glucose levels is required, and different insulin or combination therapies should be selected based on changes in the patient blood glucose. Insulin aspart is an insulin analogue that absorbs more quickly and takes effect faster than regular insulin while having a lower risk of both nocturnal and severe hypoglycemia.^[59]^ In clinical practice, insulin is often combined with AGIs to treat HD. α-glucosidase inhibitors help inhibit α-glucosidase activity and lower the rate of starch hydrolysis into glucose, while insulin promotes glucose metabolism, which means these 2 therapies complement each other by decreasing glucose production while accelerating its metabolism.^[60]^

#### 1.2.4. Treatment for chronic hepatitis.

In clinical settings, it is necessary to control the primary liver disorders to achieve better results while managing HD. The etiology of chronic liver disease is multifarious, including chronic hepatitis B, chronic hepatitis C, alcoholic or nonalcoholic liver disease, autoimmune hepatitis, and others. Consequently, different medication regimens are required based on the clinical characteristics of each type of hepatitis. Notably, given the significant correlation between hepatitis virus, liver damage, and insulin resistance, antiviral therapy can enhance liver cell function and regulate insulin resistance, thereby controlling blood glucose levels.^[61]^

##### 1.2.4.1. Antiviral therapy.

Hepatitis B virus infection poses a major global public health challenge and is a primary cause of liver cirrhosis and liver cancer. The most effective approach to managing chronic hepatitis involves maximizing the elimination of the hepatitis virus and restraining its replication. Entecavir is a first-line antiviral drug that boasts strong antiviral ability and low resistance.^[62]^ A study from China has demonstrated the efficacy of entecavir in enhancing liver fibrosis indices, suppressing viral DNA replication, regulating blood glucose levels, and improving liver functions in patients with hepatitis B cirrhosis complicated by HD.^[63]^ While nucleoside drugs such as entecavir can engender rapid antiviral effects, they cannot inhibit the emergence of liver cirrhosis. Effective antifibrotic medications are necessary to halt the progression of chronic hepatitis fibrosis.^[64]^

##### 1.2.4.2. Antifibrotic therapy.

For some hepatitis patients who cannot receive causal treatment or patients whose liver fibrosis persists or worsens even after eliminating the causative agent, antifibrotic drugs may be prescribed. Traditional Chinese medicine (TCM) is crucial in antifibrotic treatment.^[65]^ Clinical studies have revealed that in addition to antiviral drugs, the use of compound soft-shell turtle liver tablets can further reduce liver fibrosis in patients with chronic liver disease.^[66]^ Recently, some researchers have suggested that targeted TCM formulations play an essential role in treating liver fibrosis. Targeted formulations can enhance the solubility and bioavailability of TCM extracts and extract antifibrotic medications and blood glucose-lowering drugs together to achieve better treatment outcomes.^[67]^

#### 1.2.5. Surgical treatment.

Liver transplantation has been shown to be an effective measure to improve the prognosis of HD patients. However, currently, there are no clinical guidelines that include HD as an indication for liver transplantation surgery. In order to evaluate glucose homeostasis, Shetty et al measured blood glucose levels before and after transplantation surgery in 26 patients awaiting liver transplantation. The results indicated that 65% of the patients did not exhibit significant abnormalities in glucose homeostasis, whereas 23% of the patients met the American Diabetes Association criteria. Additionally, it was observed that all patients had normal HbA1c levels after transplantation.^[68]^ Maria et al^[62]^ reported that liver transplantation in HD patients, combined with treatment with tacrolimus, resulted in the cure of secondary diabetes while maintaining β-cell function.^[69]^ Hence, liver transplantation can rapidly reverse the reduction of glucose tolerance associated with cirrhosis and serves as a necessary treatment option for HD when appropriate.

However, it is pertinent to note that new-onset diabetes is one of the most common complications that arise after solid organ transplantation, resulting in negative effects on long-term survival and kidney function. This, in turn, may lead to a decline in organ function or even death.^[70,71]^ It is worth mentioning that HD patients who undergo transplantation not only need to take immunosuppressive drugs but also hypoglycemic drugs. Nevertheless, as some of these drugs have common substrates, their potential interactions cannot be ignored, and a choice needs to be made while selecting them. When using glucocorticoids for immunosuppression after liver transplantation, it is essential to take into account that glucocorticoids may reduce the body's sensitivity to glucose and impair pancreatic islet secretion function, causing insulin resistance and worsening T2DM. Hence, it is advisable for HD patients to avoid postoperative use of hormone drugs for immunosuppression.^[72]^

## 2. Conclusion

Hepatogenous diabetes as a secondary complication of liver disease, currently lacks established clinical guidelines for diagnosis and treatment. The principle of managing HD is based on treating the primary liver disease while controlling diabetes as a secondary consideration. Combination therapy involving antiviral drugs, anti-liver fibrosis drugs, and diabetes medications can be used to treat the primary liver disease and control diabetes simultaneously, thereby reducing complications and improving the prognosis of patients. Traditional Chinese medicine also plays a vital role in the treatment of primary liver diseases. Targeted delivery of TCM to the liver through technological means is a promising research direction.

In developing nations, such as China, where there is a high prevalence of hepatitis B, a considerable number of patients suffer from liver cirrhosis due to inefficient disease control measures. This scenario significantly impacts the patients’ quality of life, highlighting the need to enhance the examination system and streamline the diagnostic process. Additionally, early intervention and treatment for hepatitis B are necessary. Hepatogenous diabetes accounts for a substantial proportion of patients with liver cirrhosis and diabetes. Although liver disease itself can lead to diabetes, HD is often overlooked, lacking clinical guidelines for effective management. Therefore, individualized treatment and accurate differentiation between HD, primary diabetes, and diabetic liver disease are required for effective clinical management of HD.

The gradual deepening of research on primary diabetes is leading to the development of new generations of hypoglycemic drugs, which are entering clinical trials. When introducing these new drugs into the clinical management of HD patients’ blood glucose control, their relationship with the liver should be further considered to avoid overlooking the underlying causes of the disease. Due to the increasing amount of research being conducted on HD, it is expected that there will be significant breakthroughs in the management of this neglected disease in the future.

## Author contributions

**Conceptualization:** Yanru Deng, Keyu Li, Wen Hu.

**Investigation:** Keyu Li.

**Methodology:** Keyu Li.

**Writing – original draft:** Yanru Deng.

**Writing – review & editing:** Ang Li, WeiMing Hu, Wen Hu.
